# cGMP modulates stem cells differentiation to neurons in brain in vivo pathological implications

**DOI:** 10.1186/1471-2210-11-S1-O29

**Published:** 2011-08-01

**Authors:** Ulises Gómez-Pinedo, Regina Rodrigo, Omar Cauli, Andrea Cabrera-Pastor, Sonia Herraiz, Jose-Manuel Garcia-Verdugo, Begoña Pellicer, Antonio Pellicer, Vicente Felipo

**Affiliations:** 1 Laboratory of Cell Morphology, Centro de Investigacion Principe Felipe, Valencia, Spain; 2 Laboratory of Neurobiology, Centro de Investigacion Principe Felipe, Valencia, Spain; 3 Fundacion IVI, Instituto Universitario IVI, Universidad de Valencia, Spain; 4 Instituto Cavanilles, Universidad de Valencia; 46071 CIBERNED, Valencia, Spain

## Introduction

During brain development there is a strict control of the proliferation, migration and differentiation of neural stem cells to different cell types. Alterations in the control of these processes may result in altered balance in the formation of different cell types resulting in a long-lasting impairment of cerebral processes. This occurs for example if brain is exposed to alcohol during key stages of development which results in accelerated glial cells formation, impaired neuron formation and impaired cognitive function.

The molecular mechanisms modulating differentiation of neural stem cells to neurons or non neuronal cells are not well known. Nitric oxide (NO) plays a relevant role in this process. NO increases the activity of soluble guanylate cyclase and is a main modulator of cGMP levels in brain. It has been proposed that cGMP-mediated NO signalling may be involved in the early differentiation events of embryonic stem cells. If this is the case, pathological situations in which the production of cGMP is altered during brain development could lead to altered differentiation of stem cells to neurons or glial cells, resulting in cognitive impairment in the children. Moreover, normalizing cGMP levels in these situations could prevent the alterations in neural stem cells differentiation and cognitive impairment.

## Aims

The aims of this work were to assess in rats in vivo whether: 1) reduced cGMP levels during brain development alters the differentiation of stem cells to neurons or non neuronal cells in vivo; 2) reduced brain cGMP levels during brain development leads to cognitive impairment when the rats are young and 3) restoration of cGMP levels prevents the alterations in neural stem cells differentiation and in cognitive function.

## Methods

To assess the role of cGMP in neural stem cell differentiation we used a rat model in which brain cGMP levels are reduced during brain development. To reduce cGMP in brain of the foetuses we treated pregnant rats with nitroarginine-methylester (NAME), an inhibitor of nitric oxide synthases. We choose this treatment because, in addition to reducing the formation of nitric oxide (NO) and, subsequently, of cGMP, it is used as a model of pre-eclampsia in rats. To restore cGMP levels in rats treated with NAME, some of the rats were treated also with sildenafil, an inhibitor of cGMP-degrading phosphodiesterase. Treatment with sildenafil restores brain cGMP levels in other pathological situations associated with reduced cGMP in brain. Rats treated with NAME alone have therefore deficits in NO and cGMP formations, while rats treated with NAME and sildenafil have deficits in NO formation but not in cGMP levels.

## Results

We assessed whether the pups born from the rats treated with NAME show altered neural stem cells differentiation in prefrontal cortex and cognitive impairment at 2 months of age. We also tested whether treatment with sildenafil restores cGMP levels and prevents the changes induced by exposure to NAME.

Treatment with NAME reduced cGMP in cerebral cortex to 46% of control rats when the foetuses were on gestational day 17. Co-treatment with sildenafil completely normalized cGMP in cerebral cortex.

Reducing cGMP during brain development leads to reduced differentiation of stem cells to neurons and increased differentiation to non neuronal cells. In control rats 48% of the neural stem cells proliferating in gestational day14 differentiate to neurons and 52% to non-neuronal cells. In rats exposed to NAME only 24% of neural stem cells differentiate to neurons and 76% to non-neuronal cells. This alteration was substantially corrected by sildenafil, in rats exposed to NAME + sildenafil, 40% of neural stem cells differentiate to neurons and 60% to non-neuronal cells.

Reducing cGMP during brain development also resulted in reduced learning ability when the rats were 2 months-old. The ability to learn a Y maze conditional discrimination task was impaired in rats exposed to NAME, which needed 73 ± 6 trials to learn the task while control rats needed 54 ± 3 trials. Learning ability was not impaired in rats exposed to NAME + sildenafil or to sildenafil alone, which needed 51 ± 3 and 55 ± 5 trials, respectively, to learn the task.

## Conclusions

These results support that cGMP modulates the fate of neural stem cells in brain in vivo. High cGMP levels promote its differentiation to neurons while reduced cGMP levels promote differentiation to non neuronal (mainly glial) cells.

Situations in which cGMP levels are reduced during brain development result in reduced formation of neurons, increased formation of non neuronal (mainly glial) cells and impaired cognitive function. Restoring cGMP levels normalizes neural stem cells differentiation, formation of neurons and cognitive function.
